# *Clostridium butyricum* alleviates LPS-induced acute immune stress in goats by regulating bacterial communities and blood metabolites

**DOI:** 10.3389/fimmu.2023.1099186

**Published:** 2023-01-23

**Authors:** Chengrui Zhang, Tingyi Hou, Jihong Wang, Qingyuan Yu, Yonggen Zhang, Yukun Sun

**Affiliations:** College of Animal Science and Technology, Northeast Agricultural University, Harbin, China

**Keywords:** *Clostridium butyricum*, immune stress, microbiota, metabolites, antioxidant, goats

## Abstract

The mitigation and prevention of acute immune stress are essential for livestock production. *Clostridium butyricum* (*C. butyricum*) has shown positive effects in stabilizing intestinal microbiota disorders, improving immune function and inhibiting disease development, but its effects on ruminants are unclear. Therefore, the current trial hypothesized that *C. butyricum* could improve goats’ immune function and antioxidant capacity by regulating bacterial communities and blood metabolism and effectively alleviating the acute immune stress induced by Lipopolysaccharides (LPS). Sixteen healthy goats were fed *C. butyricum* for 70 days, and the goats were challenged with LPS on day 71. Blood and feces were collected at 0 h and 6 h after the challenge to evaluate the effects of *C. butyricum* on their intestinal microbiota, immune function, antioxidant function, and plasma metabolites. The results showed that *C. butyricum* had no significant effect on plasma biochemical parameters at the beginning of the LPS challenge. However, supplementation with *C. butyricum* increased plasma levels of IgA, IgG, T-SOD, and T-AOC (*P* < 0.05), but *TNF-α*, *IL-6*, and MDA were decreased (*P* < 0.05). In contrast, *IL-10* showed an increasing trend (*P < 0.10)*. Rectal microbiota analysis showed that *C. butyricum* significantly increased the relative abundance of Epsilonbacteraeota at the phylum level of goats; at the genus level, the relative abundances of *Campylobacter* and *Anaerorhabdus]_furcosa_group* were also significantly increased (*P < 0.05*). *Christensenellaceae_R-7_group* as the dominant microbiota also showed a significant increase in their abundance values, while *Clostridium* and *Lachnospiraceae_UCG-001* were significantly lower (*P < 0.05*). When the LPS challenge continued up to 6 h, dietary supplementation with *C. butyricum* still resulted in significantly higher plasma concentrations of IgA, *IL-10*, and T-SOD in goats than in the control group, reducing *TNF-α* levels (*P < 0.05*). In addition, plasma levels of T-CHOL and LDL were significantly reduced, and the expression of d-proline was significantly upregulated according to metabolomic analysis (*P < 0.05*). In conclusion, dietary supplementation with *C. butyricum* helped optimize the expression of bacterial communities and plasma metabolites to enhance the ability of goats to alleviate acute immune stress.

## 1 Introduction

To pursue the production goals of fast-fattening and high-level milk production, using high-concentrate diets has become an important means to improve the production performance of ruminants (1). However, in the gradual transformation of China’s animal husbandry from traditional to intensive-scale breeding, this high-concentration diet feeding model has aggravated the animal’s immune pressure and challenges. Furthermore, it reduces the animal’s immune function, giving rise to body disorders in digestion and metabolism, which can cause endotoxin immune diseases, diarrhea, and other large-scale epidemic diseases in severe cases (2). In particular, pathogenic bacteria invasion with environmental and dietary structure changes can affect the immune dysfunction of the ruminants. Nowadays, immune dysfunction has been one of the undesirable factors affecting animal production, both in grazing and intensive farming. Especially in the case of improper management and feeding, it can easily cause metabolic disorders of the organism, resulting in growth decrease and production performance and death in serious cases affecting the economic efficiency of the industry (3, 4). Currently, supplementing the diet with effective active substances such as plant extracts, probiotics, and enzymes improves immune function in ruminants (5–7). Therefore, to ensure production requirements and quality, the development of safe and effective feed additives has become a trend toward ensuring green, healthy, and high-quality animal husbandry.

*Clostridium butyricum* (*C. butyricum*) is a highly anaerobic spore that produces butyrate acid, which generally exists in nature (8). *C. butyricum* was first discovered in feces by Japanese scientists, which is highly tolerated in the gastrointestinal tract, colonizing the small intestine and distal colon, and has been used as a probiotic to regulate the human gastrointestinal environment and treat diseases (9). Meanwhile, butyric acid, a major metabolite of *C. butyricum* and one of the short-chain fatty acids (SCFA), is a nutrient necessary for maintaining intestinal health (10). It can regulate intestinal homeostasis by promoting the growth and development of intestinal cells, preventing inflammation, and improving immunity with other metabolites of *C. butyricum* (11). *C. butyricum* has been reported to inhibit the invasion of pathogens by up-regulating the expression of anti-inflammatory factors and increasing the secretion of bacteriocin in the body (12). The consumption of *C. butyricum* by patients with colitis helps maintain the dynamic balance between anti-inflammatory and pro-inflammatory cells in the body (13). Supplementation of *C. butyricum* in livestock and poultry diets can improve feed efficiency and immune function, relieve diarrhea and improve the survival of piglets (14). For piglets fed *Escherichia coli* K88 induction, it can alleviate intestinal damage by limiting detrimental inflammatory responses *via* inhibition of toll-like receptor (TLR) 2 and TLR4 signaling pathways (12). It promotes the growth and development of juvenile fish by improving intestinal development and regulating intestinal microbiota in yellow croakers (15). Therefore, dietary supplementation with *C. butyricum* could improve the health of animals to alleviate or prevent the occurrence of intestinal inflammation, which could also effectively enhance the immune function of animals.

Our previous trial’s results showed that high-concentrate dietary supplementation with *C. butyricum* could influence the production and transport of volatile fatty acid in the rumen by regulating the structure of rumen microbiota to stabilize the rumen pH and improving the persistent effect of feeding high-concentrate diets on the rumen of fattening goats in the short term (16). In the meantime, we found that *C. butyricum* could reach the intestine through the rumen and enhanced the intestinal epithelial barrier function of goats by regulating the intestinal microbial community (17). The intestinal barrier is a complex defense barrier formed by intestinal epithelial cells, mucus layer, intestinal microbiota, secreted antibodies, and other substances. Its strong immune function is crucial for maintaining normal intestinal function and ensuring the digestion and absorption of nutrients (18). Lipopolysaccharide (LPS) in ruminants is mostly secreted from the rumen or by the cell wall of infected pathogenic *Escherichia coli*, which can induce acute immune response through the systemic circulation by affecting the normal physiological function and metabolic level of the body (19). Studies have reported that exogenous LPS administration can induce acute immune responses in cattle and goats (20, 21). However, the effect of *C. butyricum* on the immune function of ruminants has rarely been reported.

Therefore, we hypothesized that *C. butyricum* could improve goats’ immune function and antioxidant capacity by modulating their intestinal microbiota and blood metabolism, effectively alleviating acute immune response or stress. Therefore, the current study investigates the effects of *C. butyricum* on blood biochemistry, inflammatory factors, immunoglobulins, antioxidant function, rectal microbiota, and plasma metabolites of goats after LPS administration. This was done by supplementing the diet of fattening goats with *C. butyricum* for a short period to assess the protective effect of *C. butyricum* on LPS-induced acute immune stress in goats.

## 2 Materials and methods

The experimental protocol (Protocol number: NEAU- [2011]-9) was approved by the Northeast Agricultural University Animal Care and Use Committee (Harbin, China). The goat-feeding experiments were conducted at the Otoki Banner family ranch (Ordos, China). The ranch meets the welfare standards of the China Animal Protection and Use Commission. Other procedures and parameters measurements were performed in the laboratory at the College of Animal Science and Technology of Northeast Agricultural University, China.

### 2.1 Animals, experimental design and diets

Sixteen six months old Albas goats raised in Otoki Banner (Ordos, China) were randomly allocated into 2 groups with 8 replicates per group. All goats were fed basal diets formulated to meet the feeding standards for meat-producing sheep and goats (NY/T 816-2004, Ministry of Agriculture and Rural Affairs, China) (22) for starter (d 1 to 14) and grower (d 15 to 71) periods. The weight of the goats was 21.9 ± 0.1kg on average, and every last goat was arranged in a separate indoor pen. Each group was given the following different treatments: Control (CON) group; goats fed a basal diet, and the *C. butyricum* (CB) group; goats fed a basal diet supplemented with 2×10^8^ CFU/kg *C. butyricum* (*C. butyricum* was provided by Hubei Lvxue Biological Industry Co., LTD, China) for 15 to 71 d. The composition and nutrient levels of diets are shown in **Table 1**. C*. butyricum* was administered directly to the goat’s mouth at the daily morning feeding to ensure its total intake. Goats were allowed to drink fresh water freely. Before the trial, all goats were dewormed with albendazole at 10.8 mg/kg of their living body weight. None of the goats had clinical symptoms of diarrhea or other diseases prior to the start of the trial.

**Table 1:** Composition and nutrient levels of diets (DM basis, %).

Ingredients	Contents	
	Concentrate	Mixed forages
Corn	40	
Corn germ meal	20	
Shotcrete corn husk	13	
DDGS	10	
Extruded soybean	8	
Molasses	3	
Limestone	4	
NaCl	1	
Compound premix^1^	1	
Total	100	
Nutrient levels^2^		
Dry matter	90.38	92.04
Crude protein	18.93	11.67
Ether extract	4.72	2.15
Crude ash	6.02	7.69
Neutral detergent Fiber (NDF)	17.58	55.75
Acid detergent fiber (ADF)	6.01	35.39
Metabolizable energy (MJ/kg)	14.09	14.05

^1^Each kilogram of composite premix includes: Ca 1.54 g, P 0.51 g, Fe 25 mg, Zn 35 mg, Cu 8 mg, Co 0.1 mg, I 0.9 mg, Se 0.25 mg, Mn 19.5 mg, VE 1000 IU, VA 3000 IU, VD 1000 IU.

^2^ME was a calculated value, while the others were measured values.

### 2.2 LPS challenge

After 71 days of the feeding experiment, four goats were randomly selected from each treatment group to respond to the LPS challenge. LPS (Escherichia coli O111:B4 Sigma, St. Louis, MO, USA) was diluted in 0.9% sterile saline to a concentration of 100 μg/ml. The goats were divided into control and treatment groups receiving 0.1 μg/kg LPS intravenously. The dosage was based on existing literature (2, 20, 21).

### 2.3 Sample collection

The total feed intake of each goat was calculated by accurately recording the amount of goat ration input and leftovers every day during the trial period. The weight of each goat was weighed before the morning feeding on days 14 and 70 of the test period, while the data were recorded to calculate the average daily gain (ADG) and feed efficiency (FCR). On the last day of the trial, the feces of goats selected for the LPS challenge were collected in cryovials and stored in liquid nitrogen until they were transferred to the laboratory and stored in a -80°C freezer for rectal microbial analysis. Blood samples (EDTA, whole blood) for all selected goats were collected from the jugular vein using 5 mL vacuum tubes (Kindly Enterprise Development Group Co., Ltd., Shanghai, China) before the morning feeding at 0 h and 6 h of LPS challenge. Baseline samples (0 h) were taken within half an hour of the LPS challenge. The blood samples were centrifuged at 3 500 g for 10 min at 4°C to obtain plasma. Plasma was collected in 2 mL centrifuge tubes and stored at −20°C to analyze plasma biochemical, antioxidant, and immune indexes. Plasma samples collected at 6 h were stored immediately in liquid nitrogen after centrifuging and transferred to the -80°C refrigerator after storage in the laboratory for untargeted metabolomics, plasma biochemical, and immune indicators analysis.

### 2.4 Plasma biochemical analysis

The plasma concentrations of total protein (TP), albumin (ALB), globulin (GLB), creatinine, blood urea nitrogen (BUN), creatinine (CREA), aspartate aminotransferase (AST), alanine aminotransferase (ALT), high-density lipoprotein (HDL), low-density lipoprotein (LDL), glucose (GLU), triglyceride (TG), and total cholesterol (T-CHOL) were analyzed using a fully automatic biochemical analyzer provided by Heilongjiang First People’s Hospital (Harbin, China).

### 2.5 Antioxidants, immunoglobulins, and inflammatory factors

The total superoxide dismutase (T-SOD), glutathione peroxidase (GSH-Px), total antioxidant capacity (T-AOC), catalase (CAT), and malondialdehyde (MDA) in plasma were determined by colorimetric analysis kits (SINO-UK Institute of Biotechnology, Beijing, China). The determination of IgA, IgG, and IgM levels was conducted by Immunoturbidimetry using the goat IgA, IgG, and IgM ELISA kits. (SINO-UK Institute of Biotechnology, Beijing, China). The concentrations of inflammatory factors tumor necrosis factor-α (*TNF-α*), interleukin-6, and -10 (*IL-6* and *IL-10*) in plasma samples were determined by commercially available goat *TNF-α*, *IL-6*, and *IL-10* ELISA kits (Nanjing Jiancheng Institute of Bioengineering, Nanjing, China).

### 2.6 Microbial composition

Following the manufacturer’s instructions, DNA from feces was extracted using the E.Z.N.A^.®^ Stool DNA Kit (D4015, Omega, Inc., USA). The total DNA was eluted in 50 μL of Elution buffer and stored at -80°C until measurement in the PCR by LC-Bio Technology Co., Ltd, Hang Zhou, Zhejiang Province, China. Universal primer sequences were designed according to the V3-V4 region of the amplified fragment: 341F (50-CCTACGGGNGGCWGCAG-30, 805R (50GACTACHVGGGTATCTAATCC-30). PCR amplification was performed in a total volume of 25 μL reaction mixture containing 25 ng of template DNA, 12.5 μL PCR Premix, 2.5 μL of each primer, and PCR-grade water was used to adjust the volume to 25 μL. The PCR conditions of amplifying the prokaryotic 16S fragments consisted of an initial denaturation at 98 °C for 30 seconds, 32 cycles of denaturation at 98 °C for 10 seconds, annealing at 54°C for 30 seconds, and extension at 72 °C for 45 seconds; and then final extension at 72 °C for 10 minutes. PCR products were confirmed with 2% agarose gel electrophoresis. Throughout the DNA extraction process, ultrapure water, instead of a sample solution, was used to exclude the possibility of false-positive results as a negative control. PCR products were purified by AMPure XT beads (Beckman Coulter Genomics, Danvers, MA, USA) and quantified by Qubit (Invitrogen, USA). The amplicon pools were prepared for sequencing while the size and quantity of the amplicon library were assessed on Agilent 2100 Bioanalyzer (Agilent, USA) and with the Library Quantification Kit for Illumina (Kapa Biosciences, Woburn, MA, USA), respectively. The libraries were sequenced on the NovaSeq PE250 platform (17).

16S rDNA were sequenced on the Illumina NovaSeq platform following the manufacturer’s recommendations provided by LC-Bio. FLASH was used to merge matching end reads. According to fqtrim (v0.94), the raw read data was quality filtered to obtain high-quality clean labels. Chimeric sequences were filtered using Vsearch software (v2.3.4). The feature table and sequence were obtained from DATA2. Alpha diversity was used to analyze the complexity of sample species diversity through Chao, Goods coverage, Shannon, and Simpson, which were calculated with QIIME2 in our sample. Beta diversity was calculated by QIIME2 and plotted by the R package. The correlation heatmap was analyzed through the Spearman correlation matrix, which was also drawn by the R package (v3.6.3). Other graphs were implemented using the R package (v3.5.2) (17).

### 2.7 Plasma metabolite analysis

The collected samples were thawed on ice, 20 μL of the sample was treated with 120 μL of precooled 50% methanol, vortexed for 1 min, incubated at room temperature for 10 min, and the extraction mixture was stored overnight at -20°C. After centrifugation at 4,000 g for 20 min, the supernatants were transferred into new 96‐well plates. The samples were stored at ‐80°C before the LC‐MS analysis. In addition, pooled QC samples were prepared by combining 10 μL of each extraction mixture. The samples were acquired by the LC‐MS system following machine orders. Firstly, all chromatographic separations were performed using an ultra-performance liquid chromatography (UPLC) system (SCIEX, UK). An ACQUITY UPLC T3 column (100 mm*2.1 mm, 1.8 µm, Waters, UK) was used for the reversed-phase separation. The column oven was maintained at 35°C, the flow rate was 0.4 ml/min, and the mobile phase consisted of solvent A (water, 0.1% formic acid) and solvent B (Acetonitrile, 0.1% formic acid). Gradient elution conditions were set as follows: 0˜0.5 min, 5% B; 0.5˜7 min, 5% to 100% B; 7~8 min, 100% B; 8˜8.1 min, 100% to 5% B; 8.1˜10 min, 5% B. The injection volume for each sample was 4 µl. A high‐resolution tandem mass spectrometer TripleTOF5600plus (SCIEX, UK) was used to detect metabolites eluted from the column. The Q‐TOF was operated in both positive and negative ion modes. The curtain gas, Ion source gas1, and Ion source gas2 were respectively set: 30, 60, and 60 PSI. The interface heater temperature was 650 °C. Ionspray voltage floating was 5000 V for the positive and ‐4500 V for the negative ion modes. The mass spectrometry data were acquired in IDA mode. The TOF mass range was from 60 to 1200 Da. The survey scans were acquired in 150 ms, and as many as 12 product ion scans were collected if exceeding a threshold of 100 counts per second (counts/s) and with a 1+charge‐state. The total cycle time was fixed to 0.56 s. Four-time bins were summed for each scan at a pulse frequency value of 11 kHz through monitoring the 40 GHz multichannel TDC detector with four‐anode/channel detection, while the dynamic exclusion was set for 4 s.

The acquired MS data pretreatments, including peak picking, peak grouping, retention time correction, second peak grouping, and annotation of isotopes and adducts, were performed using XCMS software. Intensities of each peak were recorded, and a three-dimensional matrix containing arbitrarily assigned peak indices (retention time‐m/z pairs), sample names (observations), and ion intensity information (variables) was generated. The online KEGG HMDB database was used to annotate the metabolites by matching the exact molecular mass data (m/z) samples with those from the database. Quality control‐based robust LOESS signal correction was fitted to the QC data with respect to the order of injection to minimize signal intensity drift over time. In addition, the relative standard deviations of the metabolic features were calculated across all QC samples, and those > 30% were then removed. Student t‐tests were conducted to detect differences in metabolite concentrations between the 2 phenotypes. The *P*-value was adjusted for multiple tests using an FDR (Benjamini–Hochberg). Supervised PLS‐DA was conducted through metaX to discriminate the different variables between groups. The VIP value was calculated, and a VIP cut‐off value of 1.0 was used to select important features.

### 2.8 Statistical analysis

Data were analyzed by SPSS 25.0 software (SPSS Inc., Chicago, IL) and Graphpad Prism 9 (La Jolla, CA, USA). Statistical significance of differences was performed using Student’s t tests method between groups. The test results were expressed as the mean and standard error of the mean (SEM). *P < 0.05* indicates a significant difference, *0.05 < P < 0.10* is considered a significant trend, and *P > 0.10* indicates no significant difference.

## 3 Results

### 3.1 Growth performance

**Figure 1:**
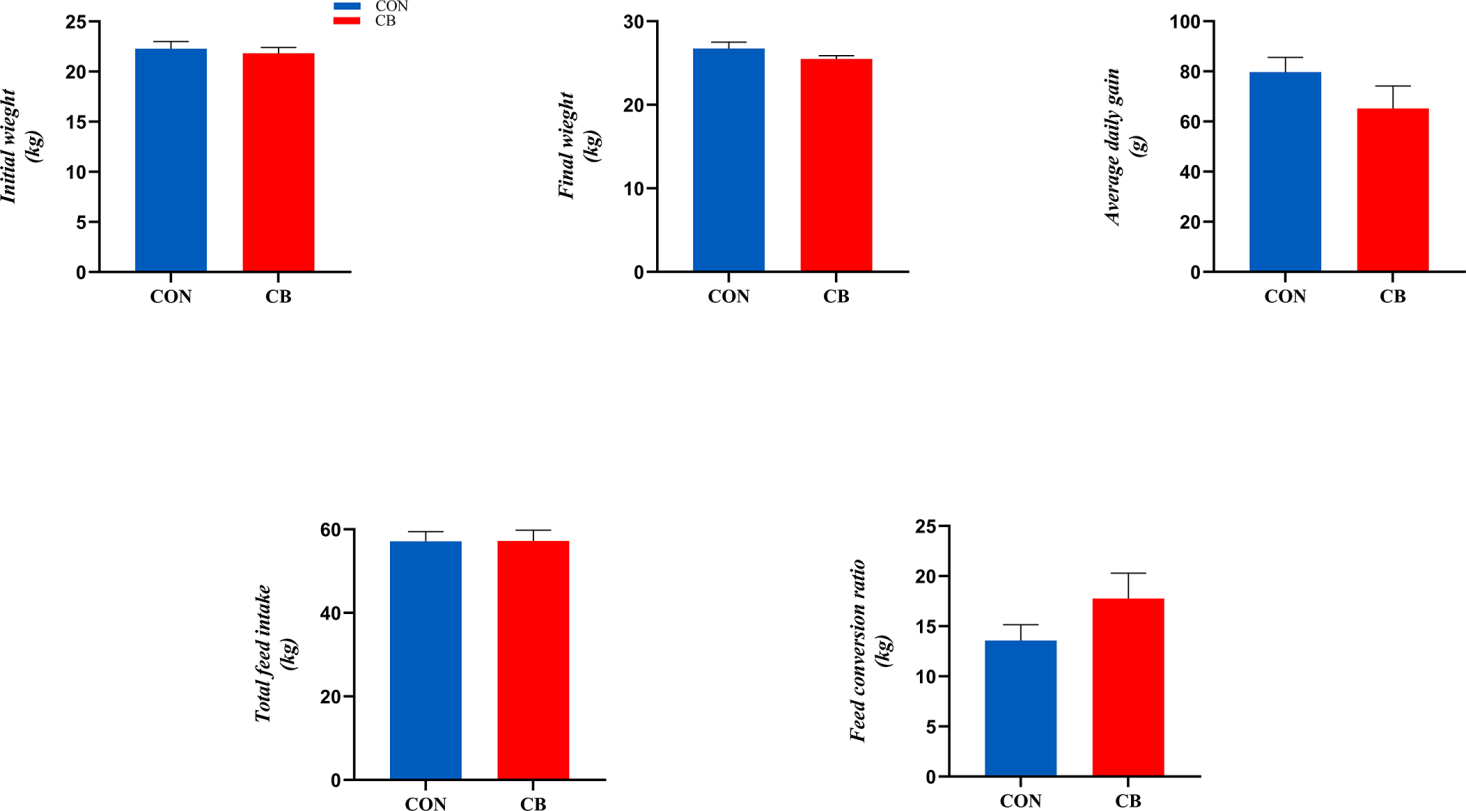
Effect of supplementation of *C. butyricum* in the diet on growth performance of goats.

The results related to growth performance are shown in **Figure 1**, where dietary supplementation with *C. butyricum* had no significant effect on goat ADG, total feed intake, and FCR (*P > 0.05*).

### 3.2 Plasma biochemical parameters

**Figure 2:**
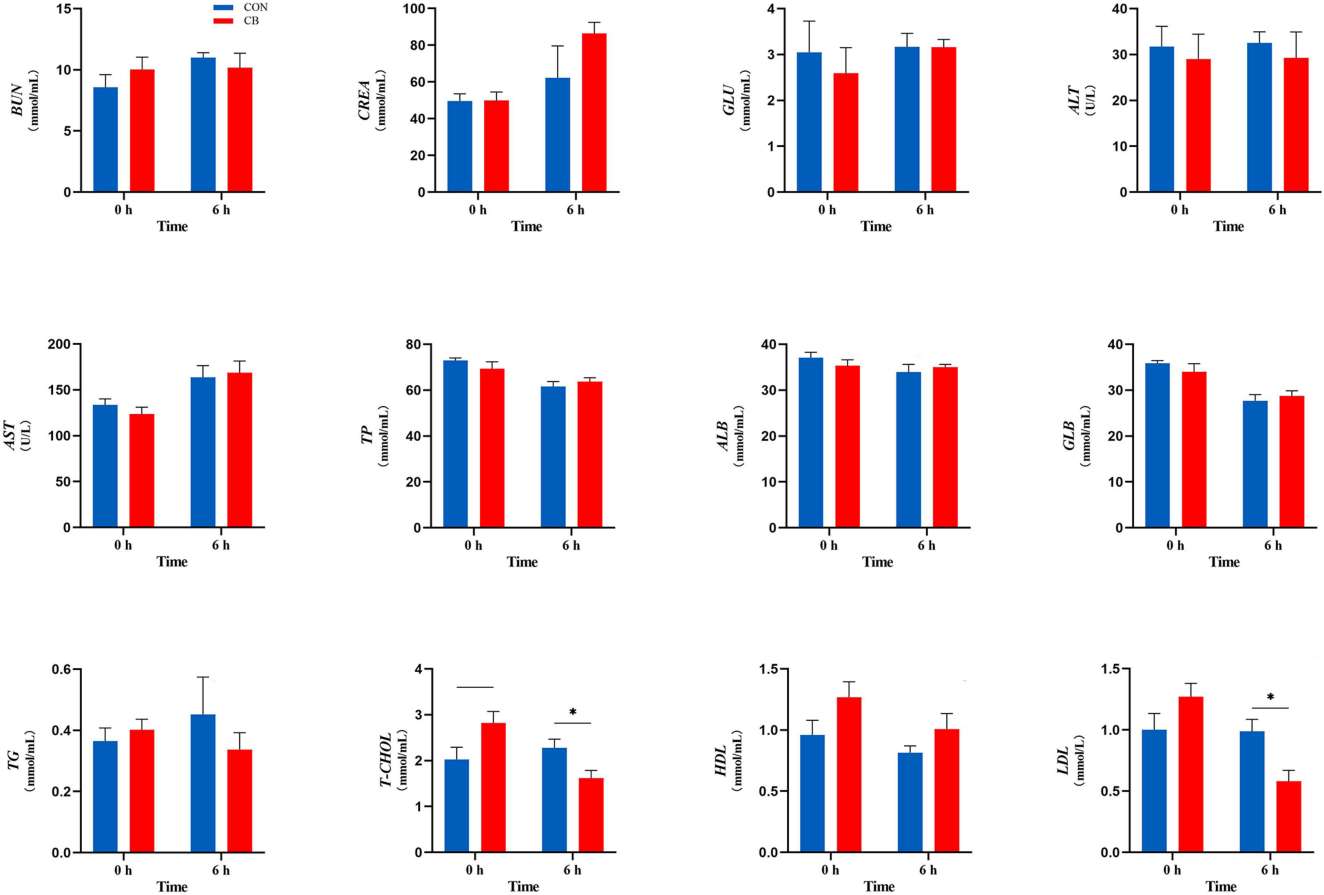
Measurement of plasma biochemical parameters at 0 h and 6 h. BUN, blood urea nitrogen; CREA, creatinine; GLU, glucose; ALT, alanine transaminase; AST, aspartate transaminase; TP, total protein; ALB, albumin; GLB, globulin; TG, triglyceride; T-CHOL, total cholesterol; HDL, high-density lipoprotein; LDL, low-density lipoprotein. *P < 0.05, —*0.05< P < 0.10* between groups.

Plasma biochemical parameters related to the LPS challenge are shown in **Figure 2**, and the concentrations of T-CHOL and LDL were affected. In the CB group, the concentration of T-CHOL showed a downward trend at 0 h (*0.05 < P < 0.10*). Meanwhile, supplementing with *C. butyricum* significantly decreased the plasma T-CHOL and LDL concentration at 6 h (*P < 0.05*). However, the other indicators of goats in the CB group at 0 h or 6 h did not change significantly after the LPS challenge (*P > 0.10*).

### 3.3 Plasma immunoglobulin and inflammatory factors

**Figure 3:**
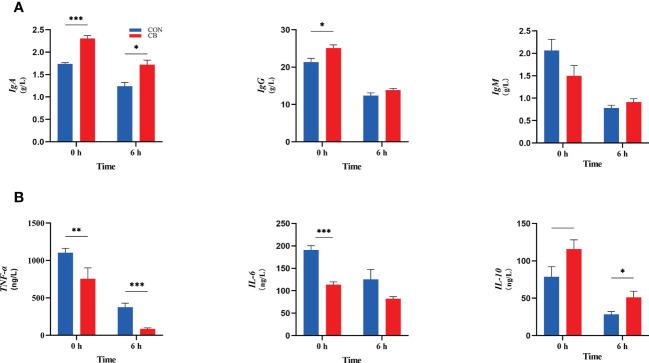
Contents of immunoglobulins and inflammatory factors in goat plasma after LPS challenge at 0 h and 6 h. **(A)** Concentrations of immunoglobulins A, G and M (IgA, IgG and IgM). **(B)** The concentration of inflammatory factors tumor necrosis factor-α, interleukin-6 and -10 (*TNF-α*, *IL-6* and *IL-10*) in plasma measured. *P < 0.05, **P < 0.01, ***P < 0.001, —*0.05< P < 0.10* between groups.

The immune indexes are shown in **Figure 3**. At 0 h, dietary supplementation with *C. butyricum* increased the plasma IgA and IgG concentrations of goats (*P < 0.05*), while there was no significant effect on IgM in the CB group (*P* > 0.10). Meanwhile, the concentrations of IgA in the CB were also higher than in CON at 6 h after the LPS challenge (*P < 0.05*). In addition, the results of immune-inflammatory factors in plasma showed that the expression of *TNF-α* and *IL-6* decreased after dietary supplementation with *C. butyricum* (*P < 0.01*). After the LPS challenge, the level of *TNF-α* of plasma in the CB group was still lower than that in the CON group after 6 hours (*P < 0.01*). *IL-10* showed an upward trend at 0 h (*0.05< P < 0.10*) but was strongly increased through the LPS challenge (*P < 0.05*).

### 3.4 Plasma antioxidant capacity

**Figure 4:**
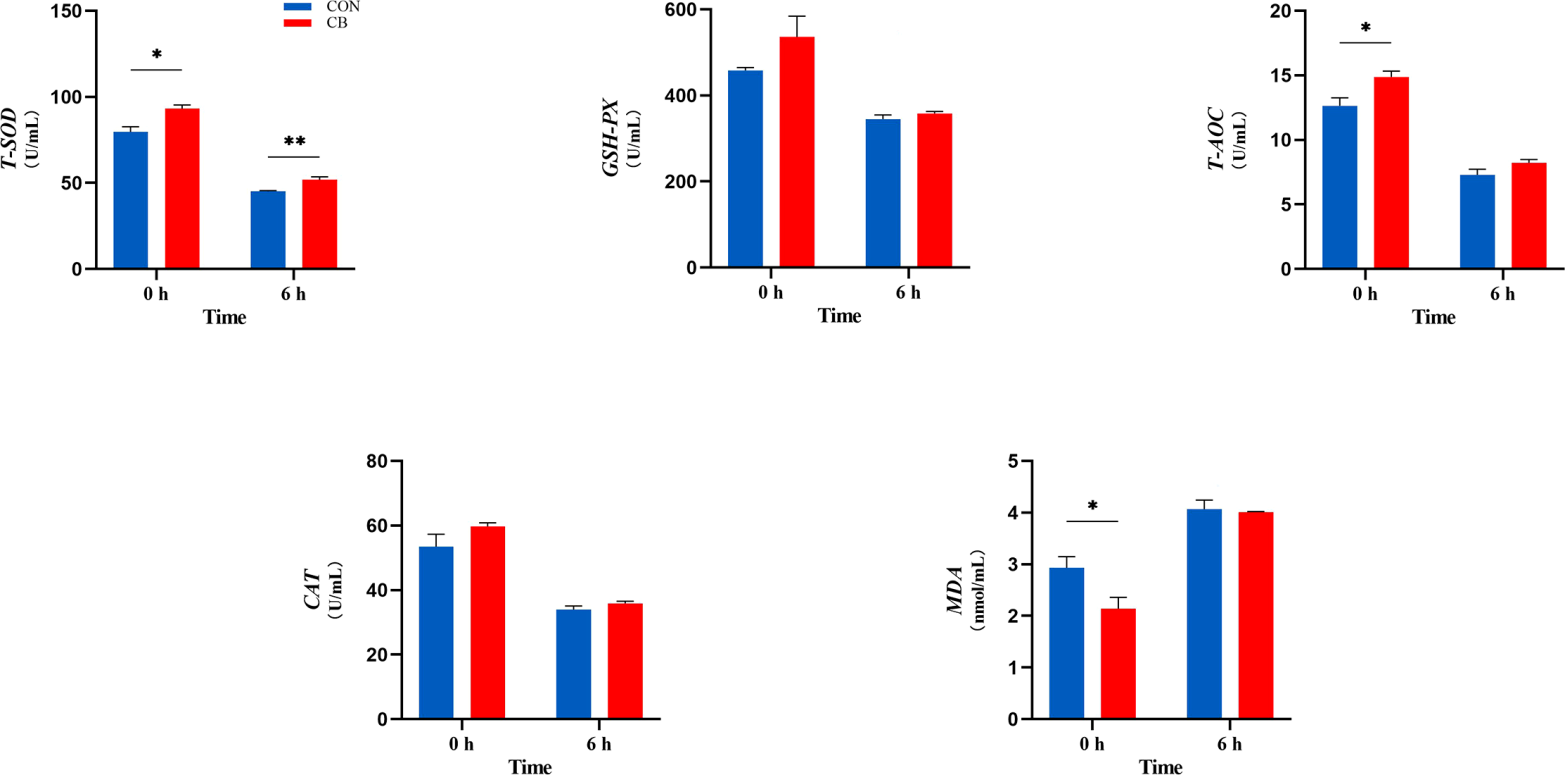
Supplement of *C. butyricum* affected the antioxidant capacity of goat after LPS challenge at 0 h and 6 h. (A) Relevant indicators of plasma antioxidant capacity: T-SOD, total superoxide dismutase; GSH-Px, glutathione peroxidase; T-AOC, total antioxidant capacity; CAT, catalase; MDA, malondialdehyde. *P < 0.05 and **P < 0.01 between groups.

In the current experiment, as shown in **Figure 4**, *C. butyricum* supplementation affected the ability of plasma antioxidants post-LPS challenge at 0 h, including T-SOD, T-AOC, and MDA (*P < 0.05*). Besides, T-SOD results in the CB group remained higher than in the CON group (*P < 0.01*). Both GSH-Px and CAT were without significant differences (*P > 0.10*).

### 3.5 Rectal microbial community

**Figure 5:**
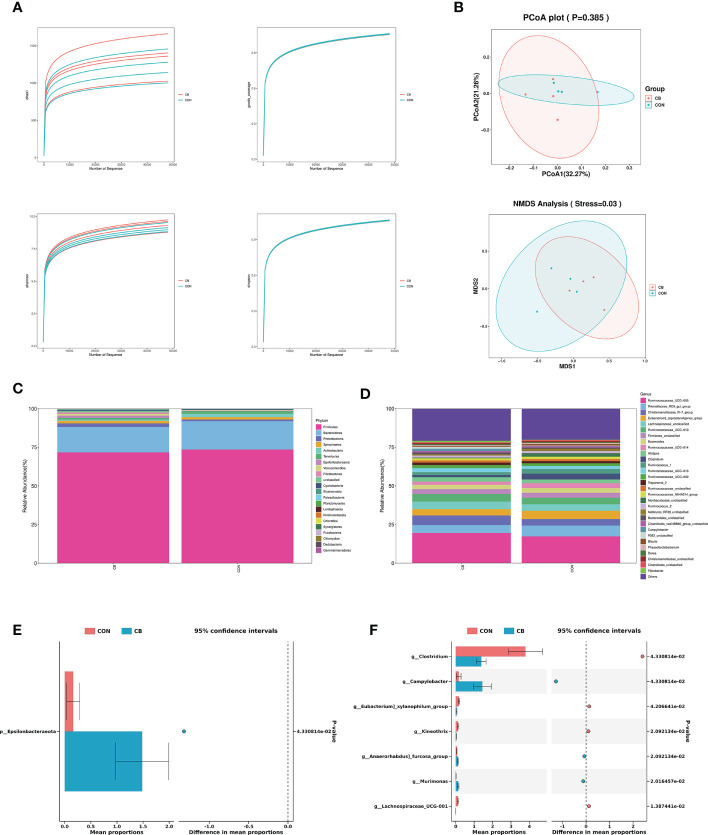
Effects of *C. butyricum* on rectal microbial diversity and richness in goats. **(A)** Alpha diversity: Chao 1, goods coverage, Shannon and simpson. **(B)** Beta diversity: PCoA and NMDS analysis. **(C)** The relative abundance of phylum level. **(D)** The relative abundance of genus level. **(E)** Significant difference in phylum level. **(F)** Significant difference in genus level.

A total of 566776 valid 16S rDNA sequences were detected in all samples. After filtering, it was found that the data quality ≥ Q20 in the valid data was better than 96.0%, indicating that the sequencing depth of the rectal microbiota content analysis was good. All sample data values were within the normal range, as shown by the alpha diversity dilution curves (**Figure 5A**). PCoA and NMDS analysis are used as one of the common methods for β-diversity analysis. In the present trial (**Figure 5B**), PCoA showed that there was no significant difference in species composition between the two groups (*P>0.05*), and NMDS map analysis further improved the credibility of the similarity in species composition (*P<0.05*).

We focused on phylum- and genus-level microbes to assess rectal microbiota composition. Microbiota with a relative abundance > 0.1% in more than half of biological replicates per group were considered as identified in the present trial. The phylum-level results show Firmicutes and Bacteroidetes predominated in each group (**Figure 5C**), and the relative abundance of Epsilonbacteraeota was significantly increased in the CB group (*P<0.05*) (**Figure 5E**). The results of the genus-level analysis showed that *Ruminococcaceae_UCG-005*, *Rikenellaceae_RC9_gut_group*, and *Christensenellaceae_R-7_group* were the dominant microbiota (**Figure 5D**), with *Christensenellaceae_R-7_group* showing an elevated trend in the CB group (**Table 2**). Furthermore, we found that *Campylobacter*, *Kineothrix*, *Anaerorhabdus]_furcosa_group*, and *Murimonas* in the CB group were strongly increased (*P<0.05*). According to **Table 2** and **Figure 5F**, we also found the relative abundance of *Clostridium*, *Eubacterium]_xylanophilum_group* and *Lachnospiraceae_UCG-001* were highly decreased at the genus level (*P<0.05*).

**Table 2:** Effects of *C. butyricum* on the rectal microbiota of goats at the genus level.

Items^2^	Treatments^1^			
	CON	CB	SEM^3^	*P*-value
*Ruminococcaceae_UCG-005*	17.34	19.57	1.16	0.374
*Rikenellaceae_RC9_gut_group*	6.98	5.04	0.77	0.231
*Christensenellaceae_R-7_group*	4.21	6.24	0.54	0.051
*Eubacterium]_coprostanoligenes_group*	5.32	4.14	0.57	0.332
*Lachnospiraceae_unclassified*	4.26	4.87	0.69	0.696
*Ruminococcaceae_UCG-010*	4.17	4.80	0.76	0.709
*Firmicutes_unclassified*	3.21	3.31	0.36	0.905
*Alistipes*	2.32	2.89	0.36	0.472
*Bacteroides*	3.09	2.76	0.27	0.574
*Ruminococcus_1*	3.04	1.85	0.53	0.299
*Treponema_2*	1.36	1.55	0.44	0.849
*Ruminococcaceae_UCG-014*	3.22	2.03	0.34	0.070
*Clostridium*	3.79	1.39	0.63	0.046
*Campylobacter*	0.16	1.44	0.34	0.044
*Atopobium*	0.03	0.16	0.03	0.035
*Negativibacillus*	0.22	0.07	0.04	0.075
*Defluviitaleaceae_UCG-011*	0.04	0.11	0.02	0.077
*Anaerorhabdus]_furcosa_group*	0.06	0.13	0.02	0.034
*Murimonas*	0.02	0.13	0.03	0.035
*Eubacterium]_xylanophilum_group*	0.17	0.04	0.03	0.034
*Kineothrix*	0.13	0.03	0.03	0.073
*Lachnospiraceae_UCG-001*	0.12	0.00	0.03	0.027
*Others*	32.97	34.66	1.55	0.626

^1^Treatments: CON, control group without on the basic diet; CB, ad 2.0 × 108 CFU C. butyricum per kg of basic diet, DM basis.

^2^SEM, Standard error of the means.

**Figure 6:**
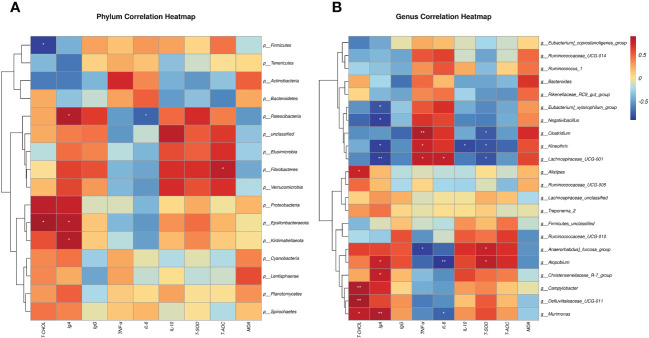
Spearman correlation between rectal microbiotas and differential plasma related indicators (0h). **(A)** Phylum correlation heatmap. **(B)** Genus correlation heatmap. Red denotes a positive correlation; blue denotes a negative correlation. The color intensity is proportional to the strength of the Spearman correlation. *P < 0.05, **P < 0.01.

Using the Spearman algorithm, we performed correlation analysis on the above-mentioned phenotypic blood indexes with significant differences (0 h) with phylum- and genus-level microbiota, respectively (**Figure 6**). According to the phylum correction heatmap (**Figure 6A**), T-CHOL and IgA performed a positive correlation with the Epsilonbacteraeota (*P<0.05*). By analyzing the genus correlation heatmap (**Figure 6B**), *Murimonas* had a certain positive correlation with T-CHOL (*P<0.05*) and IgA(*P<0.01*), but *IL-6* was the opposite (*P<0.05*). *Defluviitaleaceae_UCG-011* had a certain positive correlation with T-CHOL (*P<0.01*). In addition, this genus correlation heatmap showed that most of the microbiota with significant differences in the current trial were correlated with IgA, including *Negativibacillus*, *Kineothrix*, *Eubacterium]_xylanophilum_group* and *Lachnospiraceae_UCG-001* performed a negative correlation (*P<0.05*). *Clostridium* like *Lachnospiraceae_UCG-001* and *Kineothrix* showed a strong positive correlation with *TNF-α*, but a negative correlation with T-SOD (*P<0.05*). Meanwhile, *Clostridium* also negatively correlated with T-CHOL, IgA, *IL-10*, and T-AOC. Apart from the above, *Anaerorhabdus]_furcosa_group* also performed a positive correlation with T-SOD ((*P<0.05*), while the negative relation was with *TNF-α* and *IL-6*.

### 3.6 Plasma metabolites level

**Figure 7:**
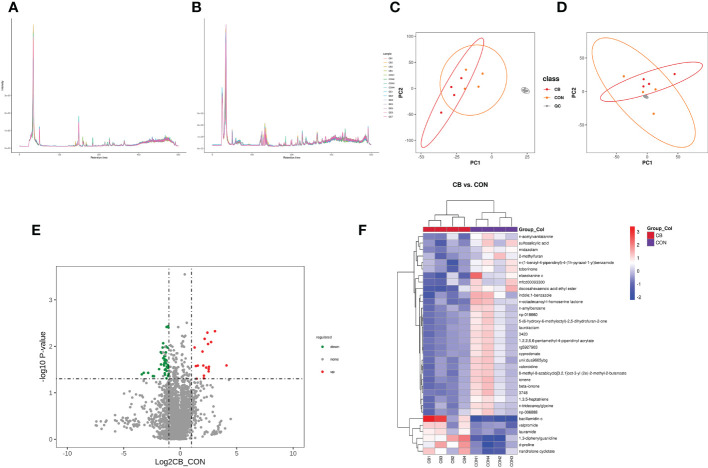
Effect of *C. butyricum* on blood metabolites in goats after LPS challenge. **(A)** Total ion chromatogram analysis chart (pos.); **(B)** Total ion chromatogram analysis chart (neg.); **(C)** PCA of the principal component (pos.); **(D)** PCA of the principal component (neg.); **(E)** Differential metabolite volcano map, the abscissa represents the difference multiple change (log2-fold change) of metabolites in different groups, and the ordinate represents the difference significance level (-log10*P*-value). Each point in the volcanic map represents a metabolite, the significantly upregulated metabolites are represented by red dots, the significantly downregulated metabolites are represented by green dots; **(F)** Cluster analysis of differential metabolites.

The total ion chromatogram demonstrates that the instrument was stable and the data results are highly reliable (**Figure 7A**: pos, **7B**: neg). Also, the PCA results of the main sample are shown (**Figure 7C**: pos, **7D**: neg). The CB vs. CON groups identified 1145 and 686 metabolites in positive and negative modes (**Table 3**), including 34 differential, 6 up-regulated, and 28 down-regulated metabolites, respectively (**Table 4** differential metabolites, *P < 0.05*). The water level plots of these 34 metabolites were shown in the volcano plot (**Figures 7, E**) and heat map (**Figure 7F**).

**Table 3:** Metabolite difference screening results.

CB vs. CON	Total Metabolite	Sig._ regulate	Sig._ up	Sig._ down
Pos.	1145	32	6	26
Neg.	686	2	0	2

**Table 4:** Differential metabolites (p < 0.05).

Names	*P*-value	Ratio	VIP	Up/Down
sulfosalicylic acid	0.0164	0.3830	2.1924	Down
n-acetylvanilalanine	0.0274	0.4868	1.9489	Down
d-proline	0.0069	4.4885	2.5005	Up
Midazolam	0.0039	0.4848	2.5082	Down
2-methylfuran	0.0281	0.4554	2.2268	Down
1,3-diphenylguanidine	0.0051	5.3088	2.6641	Up
bacillamidin c	0.0258	17.1395	2.3355	Up
elaeokanine c	0.0310	0.2738	1.7228	Down
mfcd00093300	0.0338	0.4339	1.0393	Down
valpromide	0.0499	2.7403	2.0843	Up
nandrolone cyclotate	0.0260	2.9494	2.2481	Up
docosahexaenoic acid ethyl ester	0.0134	0.3032	2.3645	Down
Lauramide	0.0106	2.4227	2.4025	Up
n-amylbenzene	0.0162	0.3984	1.1893	Down
indole;1-benzazole	0.0245	0.3080	1.2320	Down
laurolactam	0.0135	0.4155	1.1389	Down
3420	0.0200	0.3968	1.1293	Down
beta-ionone	0.0163	0.3778	1.1984	Down
unii: dua9665ybg	0.0408	0.3937	1.1079	Down
rg5927903	0.0203	0.4062	1.1417	Down
8-methyl-8-azabicyclo[3.2.1]oct-3-yl (2e)-2-methyl-2-butenoate	0.0311	0.4055	1.1123	Down
1,2,2,6,6-pentamethyl-4-piperidinyl acrylate	0.0232	0.4194	1.0973	Down
np-018660	0.0135	0.4162	1.1113	Down
cyprodenate	0.0196	0.4099	1.1428	Down
3748	0.0230	0.3791	1.1574	Down
5-(6-hydroxy-6-methyloctyl)-2,5-dihydrofuran-2-one	0.0136	0.4103	1.1223	Down
1,3,5-heptatriene	0.0038	0.4331	1.1860	Down
Ionene	0.0258	0.3718	1.2180	Down
n-tridecanoylglycine	0.0280	0.4525	1.1015	Down
valeroidine	0.0275	0.4374	1.1514	Down
np-006888	0.0429	0.4713	1.1230	Down
n-octadecanoyl-l-homoserine lactone	0.0139	0.3166	1.4068	Down
n-(1-benzyl-4-piperidinyl)-4-(1h-pyrazol-1-yl) benzamide	0.0199	0.4281	1.7016	Down
Toborinone	0.0106	0.4590	1.6264	Down

The variable important for the projection (VIP) value was used to indicate the contribution level of differential ion metabolites to the overall metabolism. The significant metabolites were sulfosalicylic acid, n-acetylvanilalanine, d-proline, midazolam, 2-methylfuran, 1,3-diphenylguanidine, bacillamidin c, elaeokanine c, mfcd00093300, valpromide, nandrolone cyclotate, docosahexaenoic acid ethyl ester, lauramide, 3420, n-amylbenzene, indole;1-benzazole, laurolactam, beta-ionone, unii: dua9665ybg, rg5927903, 8-methyl-8-azabicyclo[3.2.1]oct-3-yl (2e)-2-methyl-2-butenoate, 1,2,2,6,6-pentamethyl-4-piperidinyl acrylate, np-018660, cyprodenate, 3478, 5-(6-hydroxy-6-methyloctyl)-2,5-dihydrofuran-2-one, 1,3,5-heptatriene, ionene, n-tridecanoylglycine, valeroidine, np-006888, n-octadecanoyl-l-homoserine lactone, n-(1-benzyl-4-piperidinyl)-4-(1h-pyrazol-1-yl) benzamide and toborinone (**Table 4**, VIP ≥ 1).

**Figure 8:**
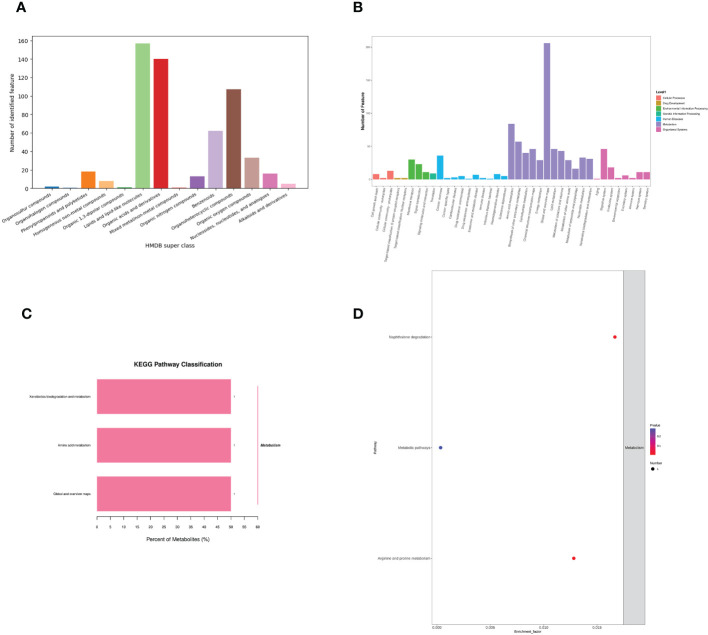
Effects of *C. butyricum* on the metabolic pathway and metabolism of goats after LPS challenge. **(A)** HMDB super class classification chart; **(B)** KEGG access notes; **(C)** KEGG pathway classification; **(D)** KEGG Enrichment Bubble Diagram.

Identification of metabolite ion annotations was made using the HMDB (human metabolome database) and KEGG (Kyoto Encyclopedia of Genes and Genomes) databases. HMDB super class classification annotation results showed that metabolites were primarily involved in lipids and lipid-like molecules, organic acids and derivatives, benzenoids, organoheterocyclic compounds, organic oxygen compounds, and other pathways (**Figure 8A**). The KEGG database was constructed to understand the functions and interactions of genes, proteins, and metabolites in biological systems (cells, tissues, etc.). Information on metabolic pathways, diseases, and organismal systems related to metabolites can be queried. The present results were mainly amino acid metabolism, biosynthesis of other secondary metabolites, chemical structure transformation maps, global and overview maps, xenobiotics biodegradation, metabolism, and other pathways (**Figure 8B**). KEGG pathway classification showed that xenobiotics biodegradation and metabolism, amino acid metabolism, with global and overview maps pathways were significantly enriched by metabolites (**Figure 8C**). The KEGG enrichment bubble diagram indicated that the main pathways were arginine, proline metabolism, and naphthalene degradation (**Figure 8D**).

**Figure 9:**
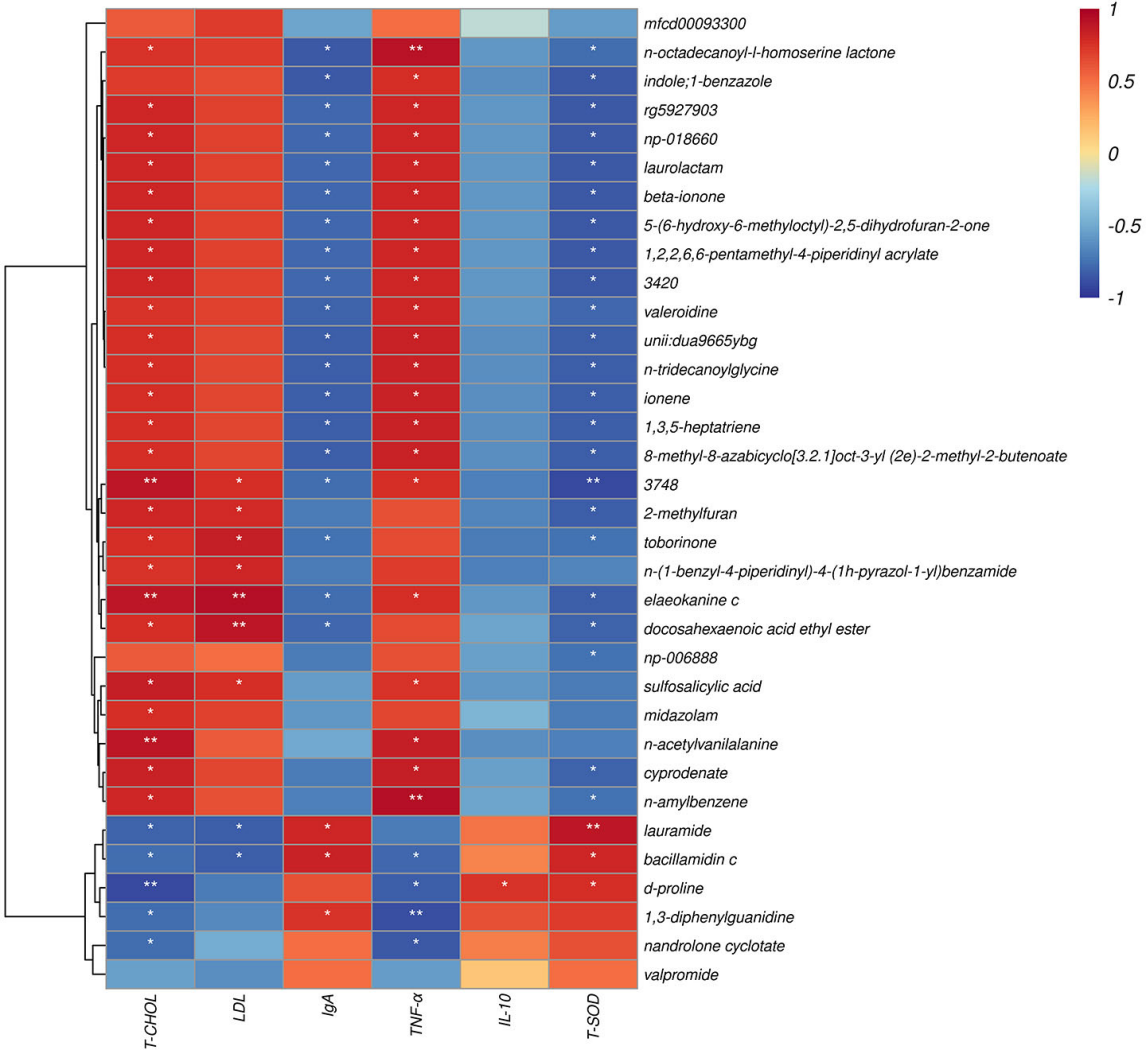
Spearman correlation between metabolite and differential plasma related indicators (6h). The abscissa is the plasma immune and antioxidant markers and the ordinate is the significant blood metabolite. Red denotes a positive correlation; blue denotes a negative correlation. *P < 0.05, **P < 0.01.

The correlation between metabolite and differential plasma-related indicators (6 h) is shown (**Figure 9**), where d-proline had a strong negative correlation with T-CHOL (*P < 0.05*). Meanwhile, d-proline also significantly correlated with other indicators, including *TNF-α*, *IL-10*, and T-SOD (*P < 0.05*). In addition, there was also a correlation between sulfosalicylic acid and T-CHOL, LDL, and *TNF-α* (*P < 0.05*). It should be noted that sulfosalicylic acid was inversely related to IgA, *IL-10*, and T-SOD, while d-proline was related to IgA’s positive correlation.

## 4 Discussion

The strong immune function of ruminants is important for maintaining their organismal health, growth, and production performance. In this experiment, at the beginning of the LPS challenge (0 h), T-CHOL in goat plasma showed a significant downward trend, while other metabolites had no significant change, which was consistent with the previous result that *C. butyricum* supplementation in fattening goat diet did not affect plasma metabolites (15). LPS-endotoxin-induced acute immune response (6 h), and we found that the contents of T-CHOL and LDL in goat plasma decreased significantly, which is consistent with the results of Jiang et al. (23). Probiotics supplementation in the diet of laying hens and calves can reduce the content of T-CHOL in plasma. In the process of acetyl CoA participating in the synthesis of T-CHOL, β-hydroxy-β-methylglutaryl CoA, as a mevalonate intermediate secreted by the liver, was inhibited by probiotics, which is positively correlated with the activity of lipase (24). In addition, the concentration of T-CHOL in the plasma is also affected by the fat composition of the diet (25). The oxidation of LDL can increase the risk of an immune response, and the generation of diseases is positively correlated with the high concentration of oxidized LDL in the blood (26). At the same time, many fermentable carbohydrates increased the production of free radicals in the host, thereby accelerating the oxidation of LDL (27). LPS is also an effective substance for establishing an animal oxidative stress model, while the high-concentrate diet was used to fatten goats in this experiment. This meant that *C. butyricum* and its metabolites could improve fat mobilization in goats to a certain extent by reducing cholesterol lipase activity and inhibiting LDL oxidation.

To our knowledge, the current trial is the first to verify the effect of dietary supplementation with *C. butyricum* on the acute immune stress of goats. Stimulated by pathogenic microbial components or metabolites, animals can actively increase the release of host-derived immunoglobulin (28). In the present trial, the plasma IgA and IgG contents of goats in the CB were higher than those in the CON at the time of the LPS challenge (0 h), and the plasma IgA concentration remained at a high level 6h later. Immunoglobulins play an important role in host-mediated humoral immunity, among which IgG is the most abundant in blood, and IgA is an antibody from external secretions (29). Studies have shown that dietary supplementation with *C. butyricum* could effectively increase piglets’ blood IgA and IgG concentrations (30). Zhang et al. (31) proved in the immune challenge test of broilers that *C. butyricum* could also improve body immunity by increasing the content of IgA and IgM in the blood. The results of the above studies were similar to the current trial, which suggested that dietary supplementation with *C. butyricum* helped to increase the plasma levels of immunoglobulins in goats. Meanwhile, immune homeostasis maintains the dynamic balance between pro-inflammatory and anti-inflammatory responses by regulating immune cells and cytokines (32). Our results revealed that after the LPS challenge with *C. butyricum* supplementation in goat diets, the concentrations of *TNF-α* and *IL-6* in plasma at 0h were significantly lower than those in the CON, and the concentration of *TNF-α* in plasma of goats in the CB remained at a low level after 6h. Surprisingly, the plasma *IL-10* concentration in the CB evolved from a relatively high trend at 0h to a significantly higher level than in the CON at 6 h after immune activation. *TNF-α* and *IL-6* are known to be cytochemokines with pro-inflammatory properties, while *IL-10* has anti-inflammatory properties. Previous studies had shown that restoration of the Treg response during immune response suppression was a key component in maintaining the balance between pro- and anti-inflammation by stimulating Treg cells to promote the secretion of *IL-10* to inhibit other antigen-presenting cells and pro-inflammatory cytokine production (28). Kashiwagi et al. (33) found that butyric acid, as the product of *C. butyricum*, promoted the differentiation of Treg cells by inhibiting histone deacetylase activity and the anti-inflammatory response of Treg cells by activating the TLR2 signaling pathway to increase the level of *IL-10* in the body. Therefore, dietary supplementation with *C. butyricum* could effectively alleviate LPS-induced acute immune stress in goats by regulating the expression of inflammatory factors and promoting the release of plasma immunoglobulins.

The invasion of exogenous pathogens or pathogenic bacteria into the organism causes an immune response, with a large number of reactive oxygen species (ROS) generated in the organism, causing oxidative stress (34). Thereby activating the glycolytic process, intensifying oxygen consumption, and making a large amount of ROS accumulate in the organism (35). Li et al. (36) reported that *C. butyricum* could reduce the oxidative stress damage induced by *Escherichia coli K88*, reversing SOD downregulation and MDA upregulation in serum by modulating the p62-keap1-Nrf2 signaling pathway. In addition, Liang (14) and Wen (37) showed that supplementing *C. butyricum* in piglets’ and pigeons’ diets also helped improve their antioxidant capacity. This was similar to the results of the current trial, where the concentrations of SOD and T-AOC in the goats’ plasma in the CB group increased significantly. MDA decreased after the LPS challenge, while the concentration of SOD remained relatively high until 6 h. Previous studies indicated that butyric acid and hydrogen, the metabolites of *C. butyricum*, could alleviate the oxidative stress level of the host by enhancing the activity of antioxidant enzymes or eliminating reactive oxygen metabolites and even interfered with the expression of fatty acid oxidase with its related genes in muscle to affect lipid metabolism (14). This was consistent with the reduction mentioned above in T-CHOL and LDL. Therefore, dietary supplementation with *C. butyricum* could also improve the antioxidant capacity of goats.

Probiotics can optimize the structure of intestinal microbiota and have a positive impact on the immune function and antioxidant properties of the body. To this end, we analyzed the rectal microbiota of goats fed *C. butyricum*. The results of this trial showed that the supplementation of *C. butyricum* in the goat diet could effectively improve the relative abundance of Epsilonbacteraeota, which was positively correlated with the decrease in plasma T-CHOL content and the increase in IgA. Studies have shown that a high-fat diet can up-regulate the abundance of Epsilonbacteraeota in the intestinal tract of mice to affect lipid metabolism, which is related to the occurrence of non-alcoholic fatty liver (38). However, Lu et al. (39) found that the relative abundance of Epsilonbacteraeota decreased significantly in the high-fat diet. After a comparative analysis of the genome of Epsilonproteobacteria, Waite et al. (40) proposed renaturalizing it into a new phylum different from Epsilonproteobacteria. It was found to have strong expressive power in the metabolism of carbon, nitrogen, and sulfur, but its specific physiological function was still unclear. It meant that Epsilonbacteraeota had a beneficial effect on the growth of goats and positively affected the improvement of lipid metabolism and immunity under the synergistic action of *C. butyricum*. This may be related to the species of the animal and its different species characteristics.

Furthermore, the genus level analysis showed that the relative abundance of *Eubacterium]_xylanophilum_group* and *Lachnospiraceae_UCG-001* in rectal bacteria decreased significantly when *C. butyricum* was added and had a strong negative correlation with IgA. *Lachnospiraceae_UCG-001* was positively correlated with *TNF-α* and *IL-6* but negatively correlated with T-SOD. Studies have shown that *Eubacterium]_xylanophilum_group* and *Lachnospiraceae_UCG-001*, both of which were *Lachnospiraceae*, could restrain the growth of short-chain fatty acid-producing bacteria in the intestinal tract and cause metabolic anomalies. It could even promote the occurrence of colon cancer (41). These results showed that adding *C. butyrate* to the diet could block the adhesion of *Eubacterium]_xylanophilum_group* and *Lachnospiraceae_UCG-001* in the intestinal tract of goats and inhibit their activity. At the same time, the correlation between the decline of *Clostridium* and *Kineothrix* relative abundance and *TNF-α*, T-SOD were consistent with previous studies in the cecum (17). It indicated that dietary supplementation of *C. butyricum* could effectively reduce the relative abundance of harmful *Clostridium* in the intestine of goats. In addition, *C. butyricum* supplementation significantly increased the relative abundance of *Anaerorhabdus]_furcosa_group*, which was negatively correlated with *TNF-α* but positively correlated with T-SOD. The relative abundance of *Christensenellaceae_R-7_group* also performed an increasing trend. *Christensenellaceae_R-7_group* helped improve the integrity of the intestinal epithelial barrier and competitively adhered to intestinal binding sites to inhibit the invasion of harmful bacteria (42). *Anaerorhabdus]_furcosa_group* could produce high-quality acetate in the intestine (43). Studies have shown that acetate supplementation in livestock and poultry diets could effectively inhibit the inflammatory response by blocking the invasion of harmful microorganisms in the intestine (44). Meanwhile, the relative abundance of *Campylobacter* and *Murimonas* increased significantly. *Murimonas* and *Christensenellaceae_R-7_group* all had a strong positive correlation with IgA. This meant that these bacteria could maintain the immunity and antioxidant capacity of the host through probiotics. *Campylobacter* was a pathogenic bacterium that has long troubled the health of poultry but had no obvious pathogenicity to other livestock (45). In the current trial, *Campylobacter* showed a significant positive correlation with the decrease of T-CHOL, which might be related to the increase of Epsilonbacteraeota abundance. David et al. (46) showed that the highly differentiated *Campylobacter* was reclassified into a variety of different genera in order to reflect its functional differences to a greater extent. Therefore, dietary supplementation with *C. butyricum* can improve the relative abundance of beneficial bacteria, optimize the intestinal microbiota of goats, and help improve their immune function and antioxidant properties.

Immune stress inhibits the growth and production performance of livestock by affecting the nutrient metabolism of the organism. The rate of catabolism of nutrients (proteins and lipids, etc.) in the organism is accelerated to enhance the synthesis of immune substances during the stress response (47). Based on the results of plasma metabolite analysis after acute immune stress in goats, this experiment found that the differential metabolite d-proline was significantly upregulated in the arginine and proline metabolic pathways. This alteration in arginine and proline metabolic pathways was associated with inflammatory responses in the organism. In the LPS challenge, arginine could effectively inhibit the expression of pro-inflammatory factors in a short time by converting to growth-promoting arginase/ornithine/polyamine and proline axis regulated by inflammatory cytokines. Meanwhile, because of the interaction of arginine metabolite itself, it was helpful to improve the defense function against antigen stimulation (48). In addition, proline had a better positive impact in scavenging free radicals and regulating the process of cellular redox reactions (49). The results of the present study showed that the significant expression of plasma inflammatory and antioxidant factors was strongly correlated with the upregulation of d-proline. This implied that supplementing goat diets with *C. butyricum* could influence the arginine and proline metabolic pathways to alleviate acute immune and stress responses induced by LPS. However, this study was only validated in a small sample size with some limitations. Further analysis is needed to elucidate the protective mechanism of dietary supplementation with *C. butyricum* on acute immune stress in goats with an increased sample size in the future.

## 5 Conclusion

**Figure 10:**
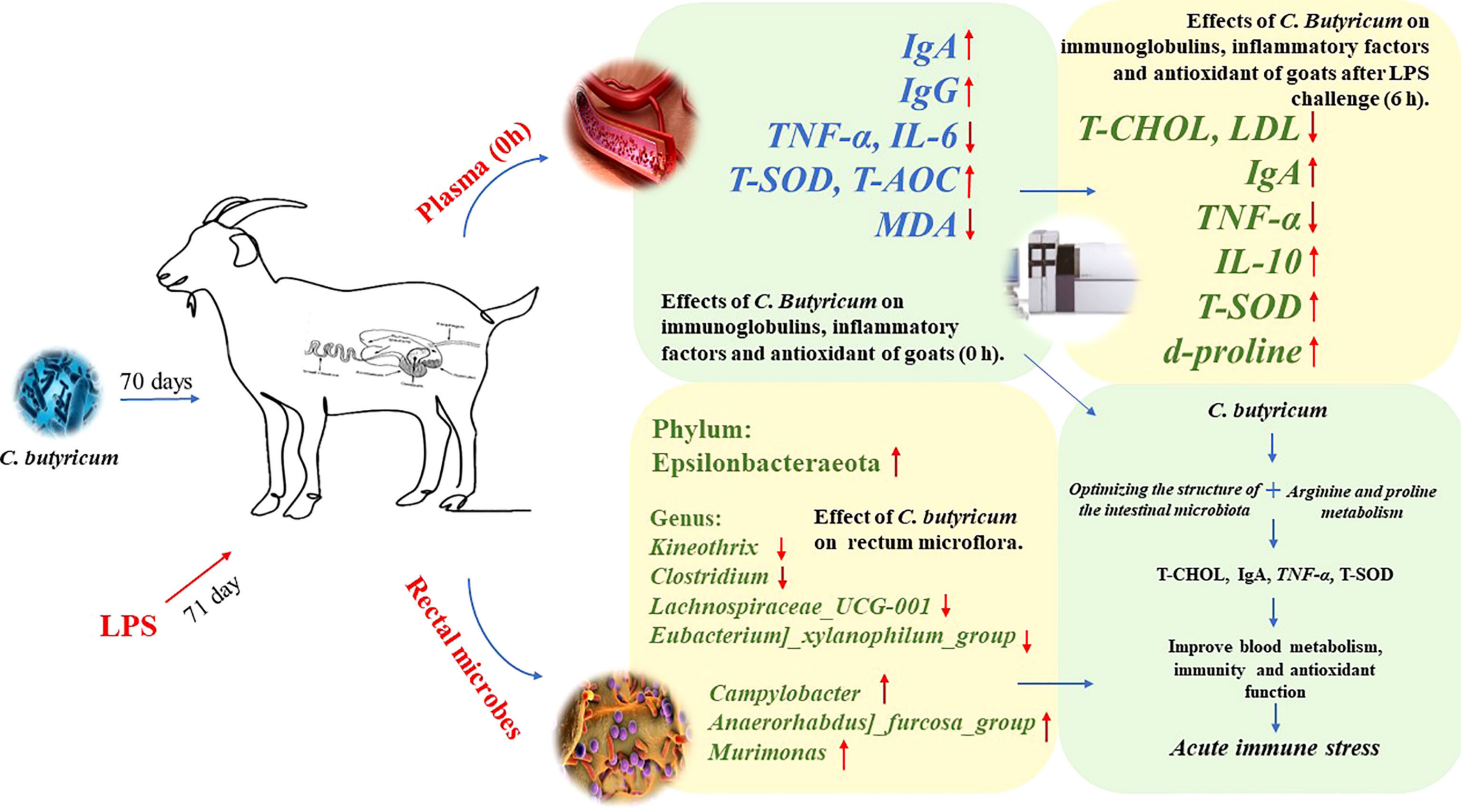
Graph abstract: *C. butyricum* alleviates LPS-induced acute immune stress in goats by regulating bacterial communities and blood metabolites.

Overall, supplementation of goat diets with *C. butyricum* in the short-term could optimize the rectal microbiota by regulating the ratio of beneficial/harmful microbiota in the rectum, thereby increasing the plasma levels of IgA, IgG, T-SOD, and T-AOC and decreasing the expressions of pro-inflammatory factors (*TNF-α* and *IL-6*) in goats. Meanwhile, dietary supplementation with *C. butyricum* improved the expressions of plasma d-proline, IgA, *IL-10*, and T-SOD, and regulated plasma lipid metabolism, which helped improve the immune system’s defense function to alleviate LPS-induced acute immune stress in goats (**Figure 10**).

## Data availability statement

16S rDNA data presented in the study are disopsited in the NCBI Sequence Read Archive (SRA) repository, accession number PRJNA913394. Metabolomics raw data have been deposited in MetaboLights. (www.ebi.ac.uk/metabolights/MTBLS6821).

## Ethics statement

The experimental protocol (Protocol number: NEAU- [2011]-9) was approved by the Northeast Agricultural University Animal Care and Use Committee (Harbin, China).

## Author contributions

CZ, YZ and YS conceptualized and designed the study; CZ conducted animal trials, analyzed data, and drafted original manuscript; TH, JW, QY performed laboratory experiments; YZ and YS reviewed and provided critical comments on the manuscript. YZ and YS were obtained the funding. All authors read and approved the final manuscript.

## References

[1] Mu C Yang W Wang P Zhao J Hao X Zhang J. Effects of highconcentrate diet supplemented with grape seed proanthocyanidins on growth performance, liver function, meat quality, and antioxidant activity in finishing goats. Anim Feed Sci Technol (2020) 266:114518. doi: 10.1016/j.anifeedsci.2020

[2] Salvesen Ø Reiten M Heegaard P Tranulis M Espenes A Skovgaard K. Activation of innate immune genes in caprine blood leukocytes after systemic endotoxin challenge. BMC Vet Res (2016) 12(1):241. doi: 10.1186/s12917-016-0870-x 27793136 PMC5084394

[3] Caroprese M Ciliberti M Santillo A Marino R Sevi A Albenzio M. Immune response, productivity and quality of milk from grazing goats as affected by dietary polyunsaturated fatty acid supplementation. Res Vet Sci (2016) 105:229–35. doi: 10.1016/j.rvsc.2016.02.018 27033938

[4] Amadori M Zanotti C. Immunoprophylaxis in intensive farming systems: the way forward. Vet Immunol Immunopathol (2016) 181:2–9. doi: 10.1016/j.vetimm.2016.02.011 26923880

[5] Li F Zhang B Zhang Y Zhang X Usman S Ding Z. Probiotic effect of ferulic acid esterase-producing *Lactobacillus plantarum* inoculated alfalfa silage on digestion, antioxidant, and immunity status of lactating dairy goats. Anim Nutr (2022) 11:38–47. doi: 10.1016/j.aninu.2022.06.010 36091259 PMC9404276

[6] Santillo A Annicchiarico G Caroprese M Marino R Sevi A Albenzio M. Probiotics in milk replacer influence lamb immune function and meat quality. Anim (2012) 6(2):339–45. doi: 10.1017/S1751731111001571 22436193

[7] Liu Z Li Y Zhao C Liu Z Wang L Li X. Effects of a combination of fibrolytic and amylolytic enzymes on ruminal enzyme activities, bacterial diversity, blood profile and milk production in dairy cows. Anim (2022) 16(8):100595. doi: 10.1016/j.animal.2022.100595 35907385

[8] Yang X Zhang B Guo Y Jiao P Long F. Effects of dietary lipids and *Clostridium butyricum* on fat deposition and meat quality of broiler chickens. Poult Sci (2010) 89:254–60. doi: 10.3382/ps.2009-00234 20075277

[9] Hagihara M Kuroki Y Ariyoshi T Higashi S Fukuda K Yamashita R. *Clostridium butyricum* modulates the microbiome to protect intestinal barrier function in mice with antibiotic-induced dysbiosis. iSci (2020) 23(1):100772. doi: 10.1016/j.isci.2019.100772 PMC6970176 31954979

[10] Zhang J Chen X Liu P Zhao J Sun J Guan W. Dietary *Clostridium butyricum* induces a phased shift in fecal microbiota structure and increases the acetic acid-producing bacteria in a weaned piglet model. J Agric Food Chem (2018) 66:5157–66. doi: 10.1021/acs.jafc.8b01253 29683328

[11] Lu J Yao J Xu Q Zheng Y Dong X. *Clostridium butyricum* relieves diarrhea by enhancing digestive function, maintaining intestinal barrier integrity, and relieving intestinal inflammation in weaned piglets. Livest Sci (2020) 239:104112. doi: 10.1016/j.livsci.2020.104112

[12] Li H Liu X Shang Z Qiao J. *Clostridium butyricum* helps to alleviate inflammation in weaned piglets challenged with enterotoxigenic escherichia coli K88. Front Vet Sci (2021) 8:683863. doi: 10.3389/fvets.2021.683863 34277756 PMC8282889

[13] Cai M Zeng L Li LJ Mo LH Xie RD Feng BS. Specific immunotherapy ameliorates ulcerative colitis. Allergy Asthma Clin Immunol (2016) 12:37. doi: 10.1186/s13223-016-0142-0 27499766 PMC4975874

[14] Liang J Raza S Kou S Chen C Yao M Wu Y. Effect of *Clostridium butyricum* on plasma immune function, antioxidant activity and metabolomics of weaned piglets. Livest Sci (2020) 241:104267. doi: 10.1016/j.livsci.2020.104267

[15] Yin Z Liu Q Liu Y Gao S He Y Yao C. Early life intervention using probiotic *Clostridium butyricum* improves intestinal development, immune response, and gut microbiota in Large yellow croaker (Larimichthys crocea) larvae. Front Immunol (2021) 12:640767. doi: 10.3389/fimmu.2021.640767 33763082 PMC7982665

[16] Zhang C Yu Q Wang J Yu Y Zhang Y Sun Y. Effects of dietary supplementation with *Clostridium butyricum* on growth performance, apparent digestibility, blood metabolites, ruminal fermentation and bacterial communities of fattening goats. Front Nutr (2022) 9:888191. doi: 10.3389/fnut.2022.888191 35685891 PMC9173004

[17] Zhang C Hou T Yu Q Wang J Ni M Zi Y. *Clostridium butyricum* improves the intestinal health of goats by regulating the intestinal microbial community. Front Microbiol (2022) 13:991266. doi: 10.3389/fmicb.2022.991266 36204609 PMC9530180

[18] He L Wang C Simujide H Aricha H Zhang J Liu B. Effect of early pathogenic escherichia coli infection on the intestinal barrier and immune function in newborn calves. Front Cell Infect mi (2022) 12:818276. doi: 10.3389/fcimb.2022.818276 PMC8900010 35265533

[19] Samarasinghe M Sehested J Larsen T Hernández-Castellano L. Oral administration of lipopolysaccharides from *Escherichia coli* (serotype O111:B4) does not induce an effective systemic immune response in milk-fed Holstein calves. J Dairy Sci (2020) 103(6):5525–31. doi: 10.3168/jds.2019-17404 32253037

[20] Benjamin A Korkmaz F Elsasser T Kerr D. Neonatal lipopolysaccharide exposure does not diminish the innate immune response to a subsequent lipopolysaccharide challenge in Holstein bull calves. J Dairy Sci (2016) 99(7):5750–63. doi: 10.3168/jds.2015-10804 27108165

[21] Liang Y Zhou J Ji K Liu H Degen A Zhai M. Protective effect of resveratrol improves systemic inflammation responses in LPS-injected lambs. Animals (2019) 9(11):872. doi: 10.3390/ani9110872 31661768 PMC6912468

[22] Wang J Lu D Yang H Yang Z Luo Q Yang Y. Ministry of agriculture and rural affairs of the people's republic of China (2004). feeding standard of meat-producing sheep and goats. (2004) Beijing: China Quality and Standards Publishing & Media Co., Ltd.

[23] Jiang X Xu HJ Cui Z Zhang Y. Effects of supplementation with lactobacillus plantarum 299v on the performance, blood metabolites, rumen fermentation and bacterial communities of preweaning calves. Livest Sci (2020) 239:104120. doi: 10.1016/j.livsci.2020.104120

[24] Sanders M. Considerations for use of probiotic bacteria to modulate human health. J Nutr (2000) 130(2):384S–90S. doi: 10.1093/jn/130.2.384S 10721912

[25] Wu J Zhou R Liu L Casper D Lang X Wang C. Growth performance, nutrient digestibility, blood parameters, and carcass characteristics by lambs fed an oregano and cobalt blend. Anim (2021) 15(10):100365. doi: 10.1016/j.animal.2021.100365 34543994

[26] Bing H Wang J Zhang C Cai H. Positive correlation between *in vivo* oxidized LDL and LDL immune complexes. Clin Biochem (2004) 37(1):72–5. doi: 10.1016/j.clinbiochem.2003.08.005 14675566

[27] Sommariva E Stadiotti I Casella M Catto V Dello Russo A Carbucicchio C. Oxidized LDL-dependent pathway as new pathogenic trigger in arrhythmogenic cardiomyopathy. EMBO Mol Med (2021) 13(9):e14365. doi: 10.15252/emmm.202114365 34337880 PMC8422076

[28] Stoeva M Garcia-So J Justice N Myers J Tyagi S Nemchek M. Butyrate-producing human gut symbiont, *Clostridium butyricum*, and its role in health and disease. Gut Micr (2021) 13(1):1–28. doi: 10.1080/19490976.2021.1907272 PMC8078720 33874858

[29] Chang M Wei J Hao L Ma F Li H Zhao S. Effects of different types of zinc supplement on the growth, incidence of diarrhea, immune function, and rectal microbiota of newborn dairy calves. J Dairy Sci (2020) 103(7):6100–13. doi: 10.3168/jds.2019-17610 32307167

[30] Wang K Cao G Zhang H Li Q Yang C. Effects of *Clostridium butyricum* and enterococcus faecalis on growth performance, immune function, intestinal morphology, volatile fatty acids, and intestinal flora in a piglet model. Food Funct (2019) 10(12):7844–54. doi: 10.1039/c9fo01650c 31793606

[31] Zhang L Cao G Zeng X Zhou L Ferket P Xiao Y. Effects of *Clostridium butyricum* on growth performance, immune function, and cecal microflora in broiler chickens challenged with *Escherichia coli K88* . Poult Sci (2014) 93(1):46–53. doi: 10.3382/ps.2013-03412 24570422

[32] Hayashi A Sato T Kamada N Mikami Y Matsuoka K Hisamatsu T. A single strain of *Clostridium butyricum* induces intestinal IL-10-producing macrophages to suppress acute experimental colitis in mice. Cell Host Micro (2013) 13(6):711–22. doi: 10.1016/j.chom.2013.05.013 23768495

[33] Ikkou K Rimpei M Takashi S Kyoko K Keita S Makoto M. Smad2 and Smad3 inversely regulate TGF-β autoinduction in *Clostridium butyricum*-activated dendritic cells. Immunity (2015) 43(1):65–79. doi: 10.1016/j.immuni.2015.06.010 26141582

[34] Muralidharan S Mandrekar P. Cellular stress response and innate immune signaling: Integrating pathways in host defense and inflammation. J Leukoc Biol (2013) 94(6):1167–84. doi: 10.1189/jlb.0313153 PMC3828604 23990626

[35] Hernandez E Talactac M Fujisaki K Tanaka T. The case for oxidative stress molecule involvement in the tick-pathogen interactions -an omics approach. Dev Comp Immunol (2019) 100:103409. doi: 10.1016/j.dci.2019 31200008

[36] Li H Shang Z Liu X Qiao Y Wang K Qiao J. *Clostridium butyricum* alleviates enterotoxigenic *Escherichia coli K88*-induced oxidative damage through regulating the p62-Keap1-Nrf2 signaling pathway and remodeling the cecal microbial community. Front Immunol (2021) 12:771826. doi: 10.3389/fimmu.2021.771826 34899723 PMC8660075

[37] Wen J Zhao W Li J Hu C Zou X Dong X. Dietary supplementation of chitosan oligosaccharide-*Clostridium butyricum* synbiotic relieved early-weaned stress by improving intestinal health on pigeon squabs (Columba livia). Front Immunol (2022) 13:926162. doi: 10.3389/fimmu.2022.926162 35844624 PMC9284028

[38] Yang X Zheng M Zhou M Zhou L Ge X Pang N. Lentinan supplementation protects the gut-liver axis and prevents steatohepatitis: The role of gut microbiota involved. Front Nutr (2022) 8:803691. doi: 10.3389/fnut.2021.803691 35127789 PMC8810540

[39] Lu L Tang M Li J Xie Y Li Y Xie J. Gut microbiota and serum metabolic signatures of high-Fat-Induced bone loss in mice. Front Cell Infect Microbiol (2021) 11:788576. doi: 10.3389/fcimb.2021.788576 35004355 PMC8727351

[40] Waite D Vanwonterghem I Rinke C Parks D Zhang Y Takai K. Comparative genomic analysis of the class epsilonproteobacteria and proposed reclassification to epsilonbacteraeota (phyl. nov.). Front Microbiol (2017) 8:682. doi: 10.3389/fmicb.2017.00682 28484436 PMC5401914

[41] Li H Liu F Lu J Shi J Guan J Yan F. Probiotic mixture of lactobacillus plantarum strains improves lipid metabolism and gut microbiota structure in high fat diet-fed mice. Front Microbiol (2020) 11:512. doi: 10.3389/fmicb.2020.00512 32273874 PMC7113563

[42] Li J Fang B Pang G Zhang M Ren F. Age- and diet-specific effects of chronic exposure to chlorpyrifos on hormones, inflammation and gut microbiota in rats. Pestic Biochem Physiol (2019) 159:68–79. doi: 10.1016/j.pestbp.2019.05.018 31400786

[43] Chen H Wang Y Zhang J Bao J. Intestinal microbiota in white spot syndrome virus infected red swamp crayfish (*Procambarus clarkii*) at different health statuses. Aquaculture (2021) 542:736826. doi: 10.1016/j.aquaculture.2021.736826

[44] Silva B Vieira F Mouri O Ferreira G Seiffert W. Salts of organic acids selection by multiple characteristics for marine shrimp nutrition. Aquaculture (2013) 384-387:104–10. doi: 10.1016/j.aquaculture.2012.12.017

[45] Edward M Mark R Amber G George L. *Campylobacter* jejuni response to ox-bile stress. FEMS Immunol Med Microbiol (2007) 49(1):165–72. doi: 10.1111/j.1574-695X.2006.00190.x 17266724

[46] Wagenberg C Horne P Asseldonk M. Cost-effectiveness analysis of using probiotics, prebiotics, or synbiotics to control *Campylobacter* in broilers. Poult Sci (2020) 99(8):4077–84. doi: 10.1016/j.psj.2020.05.003 PMC7598006 32731995

[47] Bi S Shao J Qu Y Hu W Ma Y Cao L. Hepatic transcriptomics and metabolomics indicated pathways associated with immune stress of broilers induced by lipopolysaccharide. Poult Sci (2022) 101(12):102199. doi: 10.1016/j.psj.2022.102199 36257073 PMC9579410

[48] Satriano J. Arginine pathways and the inflammatory response: interregulation of nitric oxide and polyamines: review article. Amino Acids (2004) 26(4):321–9. doi: 10.1196/annals.1304.004 15290337

[49] Wu G Bazer F Burghardt R Johnson G Kim S Knabe D. Proline and hydroxyproline metabolism: implications for animal and human nutrition. Amino Acids (2010) 40(4):1053–63. doi: 10.1007/s00726-010-0715-z PMC3773366 20697752

